# Partitioning of One-Carbon Units in Folate and Methionine Metabolism Is Essential for Neural Tube Closure

**DOI:** 10.1016/j.celrep.2017.10.072

**Published:** 2017-11-14

**Authors:** Kit-Yi Leung, Yun Jin Pai, Qiuying Chen, Chloe Santos, Enrica Calvani, Sonia Sudiwala, Dawn Savery, Markus Ralser, Steven S. Gross, Andrew J. Copp, Nicholas D.E. Greene

**Affiliations:** 1 Developmental Biology & Cancer Programme, UCL Great Ormond Street Institute of Child Health, University College London, London WC1N 1EH, UK; 2 Department of Pharmacology, Weill Cornell Medical College of Cornell University, 1300 York Avenue, New York, NY 10021, USA; 3 The Francis Crick Institute, 1 Midland Road, London NW1 1AT, UK

**Keywords:** one-carbon metabolism, folic acid, neural tube defects, spina bifida, glycine cleavage system, non-ketotic hyperglycinemia, eye, Mthfr, Gldc

## Abstract

Abnormal folate one-carbon metabolism (FOCM) is implicated in neural tube defects (NTDs), severe malformations of the nervous system. MTHFR mediates unidirectional transfer of methyl groups from the folate cycle to the methionine cycle and, therefore, represents a key nexus in partitioning one-carbon units between FOCM functional outputs. Methionine cycle inhibitors prevent neural tube closure in mouse embryos. Similarly, the inability to use glycine as a one-carbon donor to the folate cycle causes NTDs in glycine decarboxylase (Gldc)-deficient embryos. However, analysis of *Mthfr*-null mouse embryos shows that neither S-adenosylmethionine abundance nor neural tube closure depend on one-carbon units derived from embryonic or maternal folate cycles. Mthfr deletion or methionine treatment prevents NTDs in *Gldc*-null embryos by retention of one-carbon units within the folate cycle. Overall, neural tube closure depends on the activity of both the methionine and folate cycles, but transfer of one-carbon units between the cycles is not necessary.

## Highlights

• Inhibition of methionine cycle activity prevents neural tube closure, causing NTDs
• Loss of embryonic and maternal MTHFR activity does not prevent neural tube closure
• Glycine is a 1C donor to the folate cycle via the glycine cleavage system in the embryo
• Ablation of glycine cleavage causes NTDs, preventable by MTHFR inactivity or methionine

## Graphical Abstract

**Figure:**
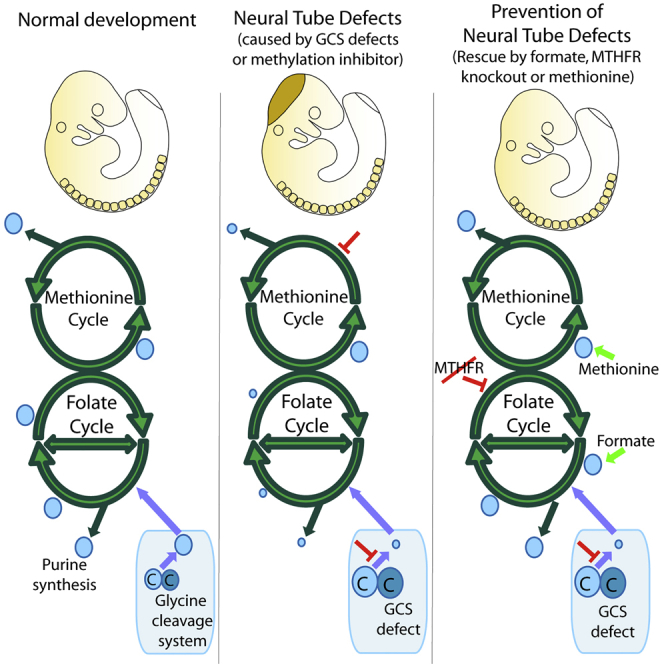

## Teaser

Leung at al. find that embryonic neural tube closure depends both on the supply of one-carbon units to the folate cycle from glycine cleavage and on the methionine cycle. In contrast, transfer of one-carbon units from the folate cycle to the methionine cycle by MTHFR is dispensable.

## Introduction

**Figure 1:**
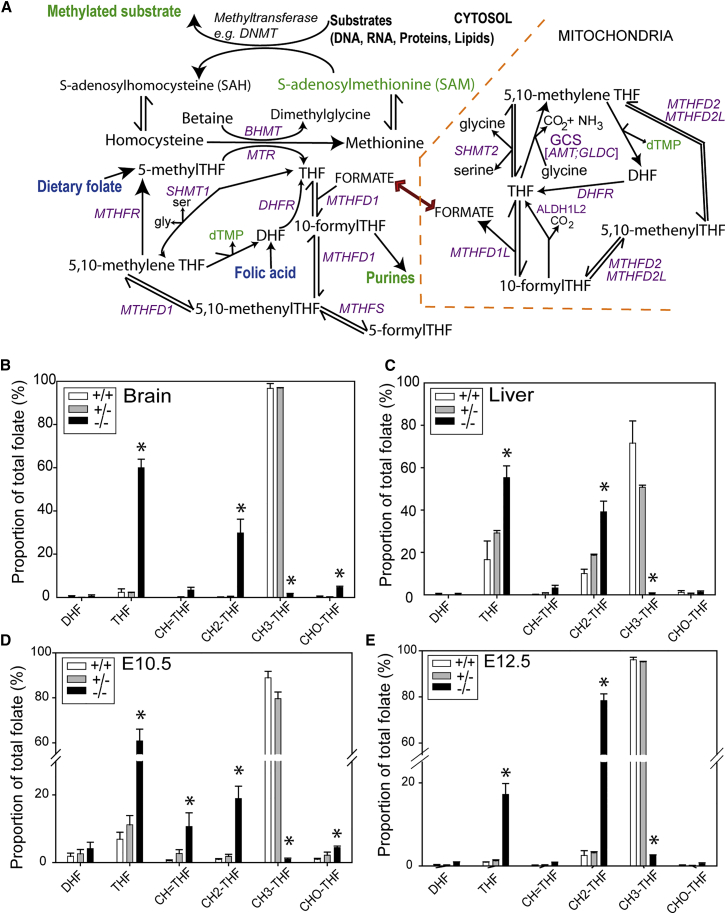
Altered Abundance of Folates in *Mthfr*-Null Embryos (A) Summary diagram of folate 1C metabolism (enzymes are indicated in purple text). (B–E) LC-MS/MS profiling of folates in (B) post-natal brain, (C) post-natal liver, and embryos at E10.5 (D) and E12.5 (E) shows that the relative abundance of 5-methyl THF (CH_3_THF) is significantly reduced in *Mthfr*^*−/−*^ (^∗^p < 0.0001 compared with *Mthfr*^*+/+*^). Conversely, the abundance of other folates was increased (^∗^p < 0.01, ^∗∗^p < 0.05; significant difference from wild-type, p < 0.05). Note that CH_2_-THF may be under-represented because of conversion to THF during analysis (maximal 20% of CH_2_-THF at pH 7 used). Number of samples, n = 3 per genotype for brain, liver, and E12.5. At E10.5, n = 4 *Mthfr*^*+/+*^, 7 *Mthfr*^*+/−*^, and 5 *Mthfr*^*−/−*^. See also Figure S1 and Table S1.

Folate one-carbon metabolism (FOCM) comprises an interlinked network of reactions that transfer one-carbon (1C) units for numerous cellular functions (Figure 1A; Tibbetts and Appling, 2010, Locasale, 2013). Key outputs include provision of 1C units for biosynthesis of thymidylate and purines and generation of S-adenosylmethionine (SAM), the “universal” methyl donor for methylation of DNA, RNA, proteins, and lipids (Brosnan et al., 2015, Ducker and Rabinowitz, 2017). FOCM is also central to a larger metabolic network through links, for example, to polyamine synthesis and creatine synthesis and the transulfuration pathway. Abnormalities of FOCM are associated with a number of diseases, including cancers, fatty liver disease, cardiovascular disease, inborn errors of metabolism (such as non-ketotic hyperglycinemia), and age-related cognitive impairment. The association of folate status with birth defects, such as neural tube defects (NTDs), also implicates FOCM in playing a key role during development.

Flux through FOCM depends on both the availability of tetrahydrofolate (THF), which is the 1C “carrier” in the folate cycle, and on supply of 1C units, principally derived from serine (Davis et al., 2004, Ducker and Rabinowitz, 2017, Yang and Vousden, 2016). Mammals cannot synthesize folates *de novo* and depend on sources from the diet and microbiota. In mice, cellular uptake of folate in the embryo is essential for viability and for completion of neural tube closure. Embryos lacking *Folr1*, encoding folate receptor 1, require supplementation with folate (methyl-THF or formyl-THF) for survival to neurulation stages and display frequent NTDs (Spiegelstein et al., 2004). In contrast, mouse embryos are relatively resistant to maternal folate deficiency. Folate depletion, achieved through defined diets and antibiotic treatment, diminishes embryonic folate content (Heid et al., 1992, Burren et al., 2008). Folate availability limits growth and developmental progression but does not dissociate these parameters or cause NTDs in wild-type embryos (Burren et al., 2008). Nevertheless, maternal folate deficiency exacerbates NTDs in some genetically susceptible mouse strains (Burren et al., 2008, Burren et al., 2010, Beaudin et al., 2011).

In parallel with a requirement for availability of the THF backbone, as supplied by dietary folate or synthetic folic acid, mouse genetic mutants provide compelling evidence that an adequate supply of 1C units is essential for neural tube closure. Serine can be a 1C donor via the action of Shmt1 in the cytoplasm and nucleus (Beaudin et al., 2011, Herbig et al., 2002, MacFarlane et al., 2011). However, the majority of 1C units entering the folate and methionine cycles appear to derive from mitochondrial FOCM, with transfer of formate to the cytoplasm (Tibbetts and Appling, 2010). The inability to generate formate from 10-formyl THF in mouse embryos lacking the mitochondrial 10-formyl THF synthetase enzyme (Mthfd1L) results in growth retardation and frequent failure of neural tube closure (Momb et al., 2013). Similarly, loss of function of components of the mitochondrial glycine cleavage system, Amt or Gldc, also causes NTDs in mice (Narisawa et al., 2012, Pai et al., 2015). Functional mutations in the corresponding human genes *AMT* and *GLDC* have also been found in NTD patients (Narisawa et al., 2012, Shah et al., 2016). Prevention of NTDs by maternal formate supplementation in *Mthfd1L* and *Gldc* mouse mutants supports the hypothesis that impaired neural tube closure results from diminished supply of 1C into FOCM in these models (Momb et al., 2013, Pai et al., 2015). This concept is supported by alteration in the relative abundance of folates and normalization of the folate profile by formate in *Gldc*-deficient embryos (Pai et al., 2015). Loss of function of the glycine cleavage system also causes an elevated embryonic tissue glycine concentration (Pai et al., 2015). It is not yet known whether the ensuing abnormalities of the folate profile and NTDs in *Gldc*-null embryos result directly from lack of glycine-derived 1C. Alternatively, it could be hypothesized that elevated glycine acts through product inhibition to reverse the action of Shmt in using serine as the 1C donor, as observed in some cancer cell lines (Labuschagne et al., 2014).

Although the *Amt*, *Gldc*, and *Mthfd1L* loss-of-function mouse mutants emphasize an essential role for supply of 1C units from mitochondrial FOCM, the relative requirements for 1C consumption in the folate and methionine cycles (Figure 1A) during neural tube closure are not well defined. Inhibitors of the methionine cycle or knockout of DNA methyltransferase cause cranial NTDs in mouse embryos (Okano et al., 1999, Dunlevy et al., 2006, Burren et al., 2008) and, similarly, inhibit neural tube closure in chick and frog embryos (Afman et al., 2005, Toriyama et al., 2017). Moreover, among folate cycle functions, impaired thymidylate biosynthesis is associated with NTDs induced by folate deficiency in *Pax3*- or *Shmt1*-null mice (Fleming and Copp, 1998, Burren et al., 2008, Beaudin et al., 2011). Therefore, various functional outputs of FOCM may be required in neural tube closure, implying a necessity for regulated flux through multiple reaction pathways for partitioning of 1C units according to metabolic need.

The transfer of 1C units from the folate cycle to the methionine cycle is mediated by 5,10-methylene tetrahydrofolate reductase (MTHFR) in a unidirectional reaction, which therefore commits 1C units to the methionine cycle (Tibbetts and Appling, 2010). Computational modeling of hepatic FOCM predicts the outcome of altered flux through specific reaction(s) (Reed et al., 2006). However, the presence of several loops within the reaction network, the potential for differential cell and tissue context-dependent regulation (Leung et al., 2013, Ducker and Rabinowitz, 2017), as well as embryonic-maternal interactions highlight the complexity in embryonic FOCM. Hence, it is necessary to analyze the effect of FOCM on neural tube closure in the developing embryo. Here we investigated the requirement for partitioning of 1C units between mitochondrial FOCM, the folate cycle, and the methionine cycle using models with loss of transfer of 1C units from the folate cycle to the methionine cycle (*Mthfr*-null) or impaired supply of 1C units to the folate cycle from mitochondria (*Gldc*-deficient).

## Results

### Methyl Groups Derived from Embryonic or Maternal Folate Metabolism Are Not Required for Neural Tube Closure

*Mthfr*-null mice are viable and born in the expected Mendelian ratio but exhibit reduced survival and a range of phenotypes, including slower growth, liver steatosis, altered brain histology, and behavioral abnormalities (Chen et al., 2001, Schwahn et al., 2003, Jadavji et al., 2015, Lawrance et al., 2011). Tissues from post-natal *Mthfr*-null mice showed diminished abundance of 5-methyl THF as a proportion of total folate (Chen et al., 2001, Ghandour et al., 2004). We extended this analysis using a liquid chromatography-tandem mass spectrometry (LC-MS/MS) methodology that allows quantification of the six major folates (Pai et al., 2015). Folates are quantified as individual polyglutamated forms, up to 7 glutamates (5 or 6 glutamates being the predominant form in mouse tissue) (Figure S1; Leung et al., 2013, Pai et al., 2015). The folate profiles confirmed that 5-methyl THF is virtually absent from the liver and brain of *Mthfr*-null mice at 3 weeks, with a corresponding increase in the relative abundance of other folates, principally THF and methylene-THF (CH_2_-THF; Figures 1B and 1C).

Among litters of embryos generated by intercrossing of *Mthfr*^*+/−*^ mice, homozygous null embryos were present in the expected Mendelian ratio, and NTDs were not observed (n = 46 *Mthfr*^*−/−*^ examined at embryonic day 9.5 [E9.5]–E12.5; Table S1), consistent with previous findings (Chen et al., 2001, De Castro et al., 2010). The major folate present in plasma is 5-methyl THF, and it is proposed that folate obtained from the maternal circulation may sustain *Mthfr*^*−/−*^ embryos *in utero*, facilitating neural tube closure and allowing survival to birth (Schwahn et al., 2004). However, folate profiling at E10.5 and E12.5 showed that *Mthfr*^−/−^ embryos contain minimal 5-methyl THF, comprising only approximately 1% or 2.5% of total folate in *Mthfr*^*−/−*^ embryos at E10.5 and E12.5, respectively (Figures 1D and 1E; Figure S1). This is comparable with a 5-methyl THF relative abundance of 89% and 95% of total folate in *Mthfr*^*+/+*^ embryos at E10.5 and E12.5 (Figures 1D and 1E) and 80% and 93% of total folate at E10.5 and E12.5 in *Mthfr*^+/−^. In *Mthfr*^*−/−*^ embryos, there was a corresponding increase in the relative abundance of other folates (Figures 1D and 1E; Figure S1).

**Figure 2:**
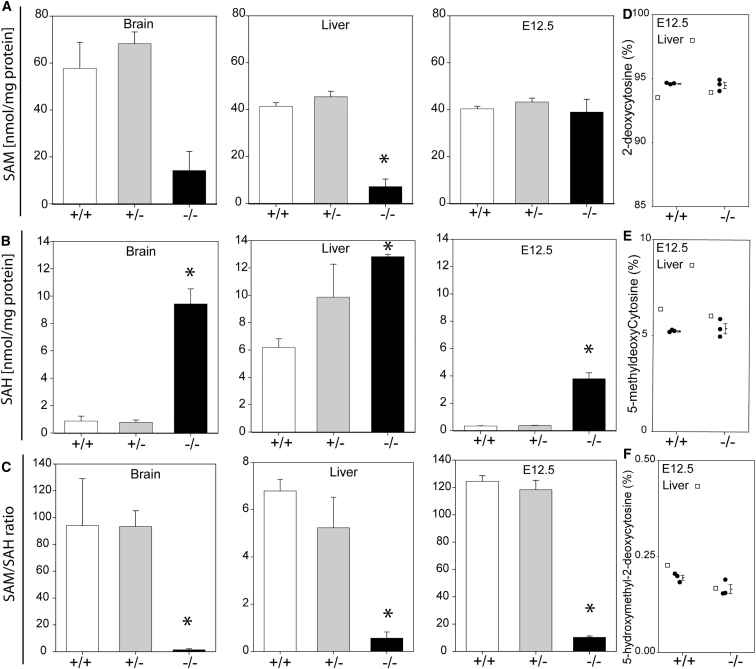
Methionine Cycle Intermediates Are Differentially Affected by *Mthfr* Genotype in Post-natal Tissue and Embryos (A) The abundance of S-adenosylmethionine (SAM) was significantly lower in livers of *Mthfr*^*−/−*^ mice than in wild-types (^∗^p < 0.05), and SAM showed a non-significant trend toward lower abundance in brains of *Mthfr*^*−/−*^ (p = 0.07). SAM abundance was not altered in *Mthfr*^*−/−*^ E12.5 embryos compared with littermates of other genotypes. (B and C) In contrast, S-adensylhomocysteine concentration (B, SAH) was elevated, and the SAM/SAH ratio (C) was reduced in *Mthfr*^*−/−*^ tissue and embryos compared with *Mthfr*^*+/+*^ (^∗^p < 0.01, significantly differs from *Mthfr*^*+/+*^). n = 3 samples per genotype for each tissue. (D–F) The proportion of (D) 2-deoxycytosine, (E) 5-methyldeoxycytosine, and (F) 5-hydroxy-2-deoxycytosine did not differ with *Mthfr* genotype among embryos at E12.5 (individual samples and mean ± SEM are shown). See also Table S2.

At post-natal stages, 5-methyl THF generated by Mthfr contributes to remethylation of homocysteine, as shown by raised plasma homocysteine concentrations in heterozygous and null mice together with an increased abundance of SAH and lower SAM in most tissues analyzed (Chen et al., 2001; Figure 2). In contrast, SAM abundance was not significantly altered in *Mthfr*^*−/−*^ embryos compared with *Mthfr*^*+/+*^ and *Mthfr*^*+/−*^ littermates (Figure 2A). Nevertheless, *Mthfr*^*−/−*^ embryos show a significantly elevated abundance of S-adenosylhomocysteine (SAH) (reflecting decreased remethylation of homocysteine) with a consequent reduction in the SAM/SAH ratio (Figures 2B and 2C). Although neural tube closure was not perturbed in *Mthfr*^*−/−*^ embryos, these findings suggest that methylation reactions could potentially be compromised. For example, in adult tissues, elevated SAH was found to be a more consistent marker of DNA hypomethylation than SAM in cystathionine β-synthase heterozygous mice maintained on a methyl-deficient diet, although whether methylation changes were detectable varied with tissue (Caudill et al., 2001). However, we found no effect of *Mthfr* genotype on cytosine methylation of DNA in the embryo (Figure 2E).

Although generation of 5-methyl THF in the embryo was not essential for neural tube closure or maintenance of SAM abundance, this did not rule out a requirement for folate cycle-derived methyl groups because null embryos developed in the context of an *Mthfr*^*+/−*^ maternal environment. We hypothesized that maternal folate-derived methyl groups could plausibly contribute to the methionine cycle. Therefore, as a further step, we carried out a two-step breeding program to generate additional litters using *Mthfr*^*−/−*^ dams. *Mthfr*^*−/−*^ offspring of *Mthfr*^*−/−*^ dams were indistinguishable from *Mthfr*^*+/−*^ littermates (n = 10 *Mthfr*^*+/−*^ and 7 *Mthfr*^*−/−*^). NTDs were not observed among these litters (Table S1), showing that embryonic and maternal Mthfr activity are both dispensable for neural tube closure. As observed for offspring of *Mthfr*^*+/−*^ dams, there was no reduction in abundance of SAM in *Mthfr*^*−/−*^ embryos from *Mthfr*^*−/−*^ dams (Table S2), analyzed at E10.5, although the SAM/SAH ratio was similarly diminished because of elevated SAH (Table S2). Methionine itself is an essential amino acid for protein synthesis. Hence, although folate-dependent methionine synthesis is dispensable for neural tube closure, it seems likely that sufficient methionine for embryo survival is obtained via the maternal diet and/or the action of Bhmt.

### Methionine Cycle Inhibitors Cause NTDs but Not via a Methyl Trap

Lack of methyl group transfer from the folate cycle to the methionine cycle does not cause NTDs, despite a significantly lower SAM/SAH ratio in *Mthfr*-null embryos. In contrast, we previously found that inhibitors of the methionine cycle (cycloleucine or ethionine) cause a high frequency of cranial NTDs in wild-type embryos in whole-embryo culture (Dunlevy et al., 2006). NTDs arise without generalized growth-retarding or toxic effects (Dunlevy et al., 2006). Cycloleucine is an inhibitor of methionine adenosyltransferase (MAT) activity that causes elevated SAH and a lower SAM/SAH ratio in treated embryos (Dunlevy et al., 2006). Ethionine is an ethyl analog of methionine that competes with methionine for MAT and is converted to S-adenosyl ethionine (SAE). Treated embryos have a significantly lower abundance of SAM, elevated SAH, and a lower SAM/SAH ratio (Dunlevy et al., 2006), whereas SAE also inhibits SAM-dependent methyltransferases directly (Alix, 1982). We cannot discount the possibility of non-specific effects, particularly of ethionine, but the induction of NTDs by two inhibitors that act via different mechanisms and without other embryotoxic effects suggests that methionine cycle function is required for neural tube closure. Two possible mechanisms could plausibly underlie NTDs induced by these inhibitors: direct suppression of methylation reactions or imposition of a “methyl trap.” The methyl trap hypothesis proposes that impaired methionine cycle flux leads to trapping of folates as 5-methyl THF because of the unidirectional nature of the Mthfr-mediated reaction. Inability to regenerate THF would suppress folate cycle functions, and this mechanism is postulated to explain some effects of vitamin B_12_ deficiency (Scott, 1999).

**Figure 3:**
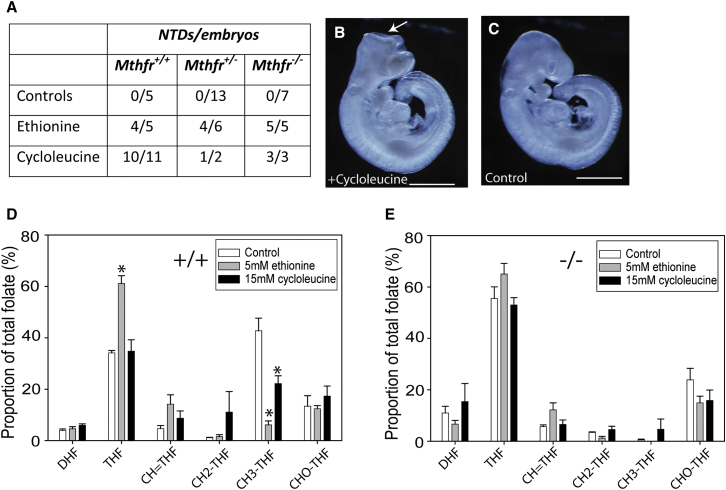
Methionine Cycle Inhibitors Cause NTDs without Imposition of a Methyl Trap (A–C) Treatment in whole-embryo culture with ethionine (5 mM) or cycloleucine (15 mM) caused NTDs among embryos of all *Mthfr* genotypes (A). NTDs affected the cranial region (open midbrain-forebrain neural folds in cycloleucine-treated embryo; arrow in B), whereas closure was completed during the culture period in vehicle-treated controls (C). Scale bar represents 1 mm. (D) In *Mthfr*^*+/+*^ embryos, ethionine treatment resulted in a significant increase in relative abundance of THF. Both ethionine and cycloleucine caused a decrease in relative abundance of 5-methyl THF compared with controls (^∗^p < 0.01, ANOVA). (E) In cultured *Mthfr*^*−/−*^embryos, abundance of folate intermediates was not significantly affected by ethionine or cycloleucine, and, as in non-cultured embryos, the relative abundance of THF and 5-methyl THF differed from *Mthfr*^+/+^ embryos (compare with D, p < 0.001, two-way ANOVA). In (D) and (E), n = 3–5 samples per genotype for each treatment

*Mthfr*-null embryos would be predicted to be resistant to a methyl trap because of their inability to generate 5-methyl THF. Arguing against a methyl trap mechanism underlying the effect of ethionine or cycloleucine, we found that NTDs were induced at similar frequencies in *Mthfr*^+/+^, *Mthfr*^+/−^, and *Mthfr*^−/−^ embryos treated in whole-embryo culture from E8.5 (Figures 3A–3C). Moreover, analysis of embryonic folate profiles showed that ethionine treatment significantly decreased the relative abundance of 5-methyl THF in *Mthfr*^*+/+*^ embryos (Figure 3D). These findings suggest that SAE inhibits Mthfr activity, in common with the strong inhibitory effect of SAM, and show that ethionine does not cause a methyl trap in wild-type embryos. In *Mthfr*^*−/−*^ embryos (which act as a methyl trap-resistant control), 5-methyl THF was virtually absent, irrespective of ethionine treatment (Figure 3E). Unlike ethionine, cycloleucine had relatively little effect on the folate profile of *Mthfr*^*+/+*^ or *Mthfr*^*−/−*^ embryos (Figures 3D and 3E). Hence, these inhibitors are proposed to cause NTDs through suppression of the methionine cycle and not depletion of 1C carrying folates through a methyl trap.

### Neural Tube Closure Depends on Glycine Decarboxylase Function

Having analyzed *Mthfr*^*−/−*^ embryos, in which partitioning of 1C units from the folate cycle to the methionine cycle is prevented, we analyzed embryos in which we hypothesize the supply of 1C units into the folate cycle is limited. Mitochondrial FOCM supplies 1C units to the cytoplasmic folate cycle as formate. This appears to be essential for neural tube closure because NTDs occur in embryos lacking expression of the mitochondrial FOCM components *Amt*, *Mthfd1L*, or *Gldc* (Narisawa et al., 2012, Momb et al., 2013, Pai et al., 2015) and may be rescued by formate supplementation. *Gldc* and *Amt* encode components of the glycine cleavage system (GCS), which mediates decarboxylation of glycine with transfer of a 1C unit to THF, generating 5,10-methylene THF (Figure 1A).

**Figure 4:**
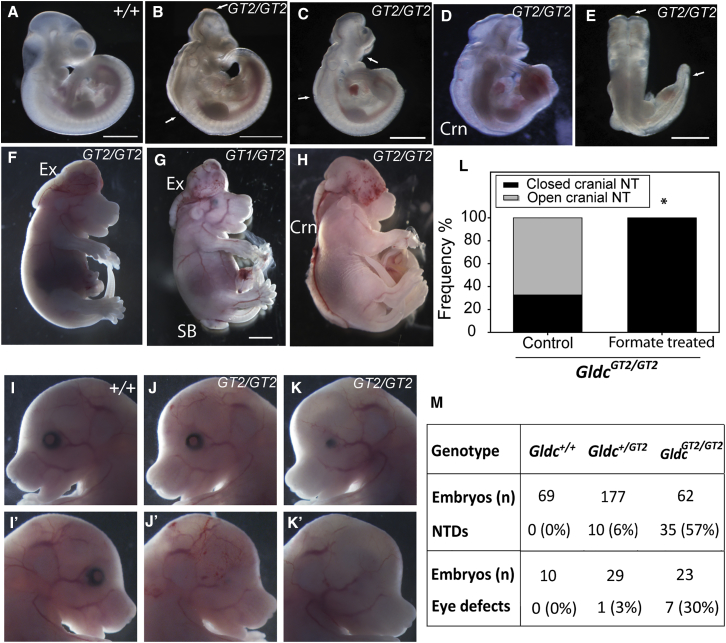
Loss of Function of the GCS Causes Neural Tube Defects (A–E) Cranial neural tube closure is complete in a *Gldc*^*+/+*^ embryo at E10.5 (A), whereas, in *Gldc*^*GT2/GT2*^ embryos (B–E), the neural folds remain open (region between arrows) in the mid-hindbrain (B), fore-hindbrain (C), or throughout the entire mid-hindbrain and spinal region (craniorachischisis [Crn], D and E). (F–H) At later stages (F–H, E15.5), failed closure in the brain, low spine, or entire brain and spine leads to the typical appearance of exencephaly (Ex, F and G), spina bifida (SB, G), and Crn (H), respectively (scale bars represent 1 mm). (I–K) Among litters examined at E16.5–18.5, unilateral (J and J’) or bilateral (K and K’) eye defects were frequently observed among *Gldc* mutant fetuses but not in the wild-type (I and I’). (L) Maternal formate supplementation prevents NTDs in *Gldc*^*GT2/GT2*^ embryos (n = 10 untreated, 9 formate-treated; ^∗^p < 0.02, Fisher’s exact test). (M) Frequency of NTDs among litters from *Gldc*^*GT2/+*^ intercrosses (n = 38 litters analyzed). Data for eye defects are included for a subset of litters analyzed at E14.5–16.5. See also Table S3.

A hypomorphic gene-trap allele of *Gldc* (denoted *Gldc*^*GT1*^) causes an approximately 90% reduction in mRNA abundance, elevated glycine concentration, and altered folate profile. NTDs arise in approximately 20% of homozygous *Gldc*^*GT1/GT1*^ embryos (Pai et al., 2015; Table S3). To analyze the effect of a greater reduction in GCS activity, we generated an additional line of *Gldc*-deficient mice (*Gldc*^*GT2*^) using an embryonic stem cell (ESC) line carrying a gene trap construct in intron 19 of *Gldc* that results in a truncated mRNA lacking exons 20–25. *Gldc* mRNA expression was undetectable in the homozygous mutant embryos by qRT-PCR. Neural tube closure was incomplete, leading to highly penetrant NTDs affecting 57% of homozygous *Gldc*^*GT2/GT2*^ mutants analyzed at E9.5–E16.5 (Figure 4; Table S3). Maternal supplementation with formate during pregnancy prevented NTDs in *Gldc*-null embryos (Figure 4L), consistent with our findings in *Gldc*^*GT1/GT1*^ hypomorphic embryos (Pai et al., 2015).

Compound mutant embryos, *Gldc*^*GT1/GT2*^, generated by intercrossing of *Gldc*^*GT1/*+^ and *Gldc*^*GT2/*+^ heterozygotes, also developed NTDs with similar penetrance (53%) as *Gldc*^*GT2/GT2*^ homozygotes. NTDs also occurred at a low frequency among heterozygous *Gldc*^*GT1/+*^ (0.7%) and *Gldc*^*GT2/+*^ (6%) embryos (Table S3). As in *Gldc*^*GT1*^ mutants, NTDs principally affected the cranial region (exencephaly) (Figures 4B, 4C, and 4F). However, in *Gldc*^*GT2/GT2*^ and *Gldc*^*GT1/GT2*^ mutants, we also observed occasional spina bifida (Figure 4G) or craniorachischisis (Figure 4H). Spina bifida results from failure to complete closure in the low spine, whereas craniorachischisis results from failure of initiation of closure at the hindbrain-cervical boundary, and the entire spinal neural tube remains open (Figures 4D and 4E). Hence, GCS function contributes to neural tube closure at all axial levels. In addition to NTDs, abnormalities of eye development occurred in 30% of *Gldc*^*GT2/GT2*^ homozygous mutants examined at E14.5–E16.5 (Figure 4M). Eye defects tended to be unilateral and resembled anophthalmia or microphthalmia (Figures 4J and 4K).

### Glycine Cleavage Provides 1C Units in Folate Metabolism and Contributes to *De Novo* Purine Biosynthesis

The lower abundance of 1C-carrying folates and rescue of NTDs by formate in *Gldc*-deficient embryos led us to hypothesize that NTDs caused by GCS disruption result from a diminished supply of glycine-derived 1C units to FOCM. On the other hand, whether there is a requirement for glycine as a 1C donor is questioned by metabolic labeling in cancer cell lines, which found that glycine cleavage does not contribute 1C units for nucleotide biosynthesis (Jain et al., 2012, Labuschagne et al., 2014, Fan et al., 2014). Instead, excess glycine led to SHMT-mediated conversion of glycine to serine and inhibition of proliferation, presumably because of depletion of 1C units that are required for nucleotide biosynthesis (Fan et al., 2014, Labuschagne et al., 2014).

Metabolic labeling in humans and in fetal lambs shows bidirectional interconversion of serine and glycine (Kalhan and Hanson, 2012, Lamers et al., 2007). In contrast to cancer cell lines, infusion of [^13^C_2_] glycine in adult humans indicated that glycine can contribute to serine synthesis via the “reverse” SHMT-mediated reaction, not only directly (as an intact molecule) but also as a 1C donor (via 5,10-methylene THF) following glycine cleavage (Kalhan and Hanson, 2012, Lamers et al., 2007, Lamers et al., 2009). Although this whole-body flux analysis confirmed that GCS activity provides 1C units for generation of 5,10-methylene THF, the majority of this is utilized in serine synthesis as opposed to other FOCM outputs (Lamers et al., 2009).

**Figure 5:**
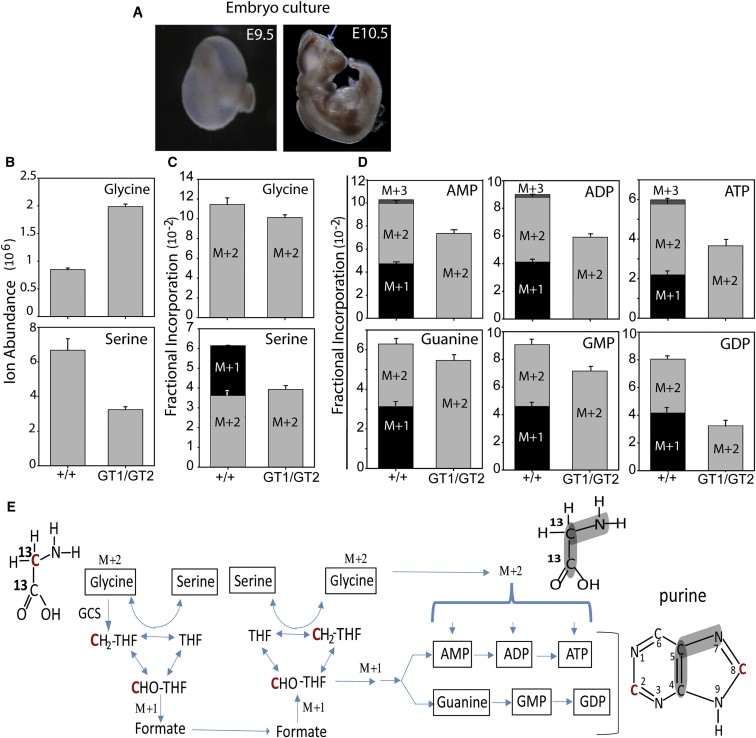
Glycine Is a 1C Donor in Neurulation-Stage Embryos (A) Labeling with [1,2-^13^C] glycine was performed in whole-embryo culture with the yolk sac intact until E10.5 (typical stage of embryos after culture is shown; the arrow indicates cranial NTD in a *Gldc*^*GT1/GT2*^ embryo). (B) The ion abundance of labeled glycine was elevated in *Gldc*-null embryos compared with wild-types (n = 5 embryos per genotype). (C–E) Fractional incorporation of labeled glycine was approximately 10% of total (C). Glycine was incorporated intact into serine (C) and purines (D and E), in which it contributes C4 and C5. In wild-type embryos, M+1 labeling of serine and purines (at C2 or C8) was also detected, implying GCS-mediated cleavage of glycine to generate 5,10-methylene THF and transfer of the 1C unit (indicated in red) via formate to formyl-THF in the cytoplasm (E). No M+1 or M+3 labeling of serine or purines was detected in *Gldc*-null embryos.

In embryos, including at neurulation stages, it has not yet been determined whether GCS activity contributes to production of 1C units for cytosolic functions of FOCM, including nucleotide biosynthesis. Given the known tissue heterogeneity in the regulation of FOCM (Brosnan et al., 2015, Ducker and Rabinowitz, 2017), we further investigated this question by labeling embryos with [1,2-^13^C] glycine or [1,2-^12^C] glycine in whole-embryo culture for a period of 24 hr from E9.5 (Figure 5A). Tissue extracts (n = 5 per genotype for each treatment) were analyzed by LC-MS.

Wild-type and *Gldc*-deficient (*Gldc*^*GT1/GT2*^) embryos exhibited uptake of labeled glycine (M+2, mass of unlabeled glycine + 2) from the culture medium (rat serum). As a proportion of total glycine, the maximum enrichment of labeled glycine was approximately 10% (Figure 5C). *Gldc-*deficient embryos contained a greater quantity of glycine, as shown by ion abundance (Figure 5B), as predicted by their lack of GCS activity (p < 0.001, t test). Some labeled glycine was converted to serine in both wild-type and *Gldc*-deficient embryos, showing that the Shmt1 and/or Shmt2 reaction is reversible in the embryo (Figure 5C). In addition to the M+2 isotopomer, a fraction of serine was the M+1 isotopomer in wild-type embryos but not in *Gldc*^*GT1/GT2*^ mutants (Figure 5C). This labeling results from transfer of a glycine-derived 1C unit from 5,10-methylene THF and confirms the activity of the GCS. Arguing against a significant reversal of the Shmt1/2 reaction toward serine production, because of excess glycine in embryos lacking GCS activity, the ion abundance of labeled serine was not elevated in *Gldc-*deficient embryos (Figure 5B). Instead, the levels showed a trend toward reduction (p = 0.054), suggesting that demand for serine as a 1C donor may be increased. Similarly, in non-cultured embryos, the elevated concentration of tissue glycine observed in Gldc^GT1/GT1^ embryos was not accompanied by an increase in total tissue serine content (Table S4).

M+1 labeling of serine in wild-type but not in *Gldc*-deficient embryos demonstrates that glycine cleavage contributes 1C units to FOCM, at least as far as 5,10-methylene THF in the mitochondria. It remained possible that all glycine-derived 1C units remain within mitochondrial FOCM, either in serine synthesis or through complete oxidation, in which 10-formyl THF is converted to CO_2_ and THF, via activity of mitochondrial 10-formyl THF dehydrogenase (ALDH1L2) (Krupenko et al., 2010). We therefore asked whether labeled glycine was utilized in *de novo* purine synthesis (Figure 5D). Intact glycine contributes C4, C5, and N7 of the purine ring in formation of the intermediate glycinamide ribonucleotide (GAR), catalyzed by Gart. In addition, C2 and C8 are contributed by 10-formyl THF (Figure 5E). Hence, purine nucleotide M+2 isotopomers can be generated from incorporation of intact [1,2-^13^C] glycine. We detected M+2 purines, including AMP, ADP, ATP, guanine, guanosine monophosphate (GMP), and guanosine diphosphate (GDP), in both *Gldc*^*+/+*^ and *Gldc*^*GT1/GT2*^ embryos (Figure 5D). M+1 and M+3 isotopomers, which can only be generated by provision of glycine-derived 1C units via 5,10-methylene THF and formate, were detected in wild-type embryos, demonstrating contribution of the GCS to *de novo* purine synthesis. We assume that the abundance of M+4 isotopomers, if formed, was below the level of detection. Notably, no M+1 or M+3 purine nucleotides were present in *Gldc*^*GT1/GT2*^ embryos. In summary, *Gldc-*deficient embryos can incorporate intact glycine into the purine ring but cannot contribute glycine-derived 1C units to purine synthesis.

### Mthfr Activity or Methionine Treatment Modifies the Frequency of NTDs Caused by Abnormal Folate Metabolism

**Figure 6:**
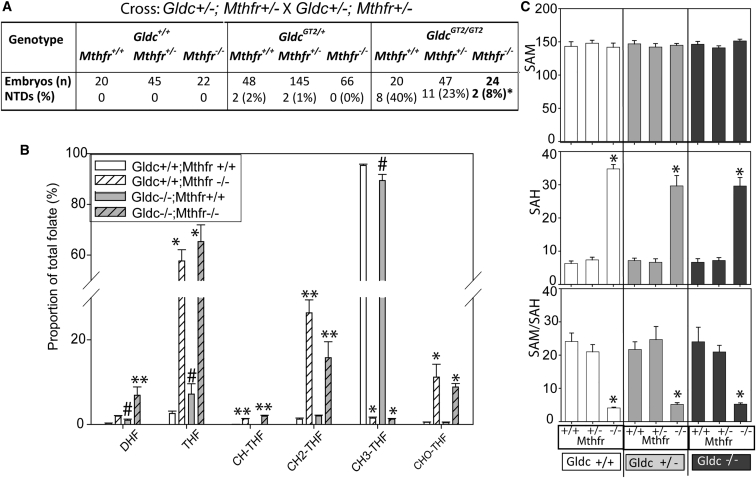
Prevention of *Gldc-*Associated NTDs by *Mthfr* Loss of Function (A) The frequency of NTDs among *Gldc* homozygous mutants is significantly affected by *Mthfr* genotype (different from *Mthfr*^*+/+*^, ^∗^p < 0.05 Fisher’s exact test). (B) Among embryos carrying loss-of-function alleles of *Gldc* and *Mthfr* (n = 4–7 samples per genotype at E11.5), we observed significant variation between genotypes in the relative abundance of each folate (p < 0001, ANOVA; Holm-Sidak pairwise comparison: ^∗∗^ indicates difference to all other genotypes, ^∗^ bars do not differ from each other but significantly differ from all other genotypes; # indicates significant difference with *Gldc* genotype in *Mthfr*^*+/+*^ embryos). (C) Abundance of SAH was significantly increased and the SAM/SAH ratio decreased in all *Mthfr*^*−/−*^embryos compared with other *Mthfr* genotypes, irrespective of *Gldc* genotype (^∗^p < 0.01, ANOVA).

In humans, the rs1801133 SNP (C677T) in *MTHFR* is associated with increased risk of NTDs in some populations (Botto and Yang, 2000), and functional mutations in *GLDC* have been identified in NTD patients (Narisawa et al., 2012). We therefore generated compound heterozygous *Mthfr*^*+/−*^; *Gldc*^*+/GT2*^ mice to examine the effects of simultaneously reducing function in both genes. Two contrasting outcomes were considered. Because methionine cycle inhibitors cause NTDs, and the SAM/SAH ratio is lower in *Mthfr*-null embryos, it appeared possible that combined loss of Mthfr and GCS activity could have an additive, deleterious effect on neural tube closure. Alternatively, we reasoned that, if insufficiency of 1C units within the folate cycle is the cause of *Gldc*-related NTDs, then prevention of 1C transfer to the methionine cycle via Mthfr could have an ameliorating effect. The double heterozygous mice were viable and were intercrossed to generate experimental litters. As in previous crosses, *Mthfr*^*−/−*^; *Gldc*^*+/+*^ embryos did not develop NTDs, whereas *Mthfr*^*+/+*^; *Gldc*^*GT2/GT2*^ embryos displayed a high frequency of cranial NTDs. Remarkably, we observed a protective effect of the *Mthfr*-null allele among *Gldc*^*GT2/GT2*^ embryos, with a significant reduction in NTD frequency among compound null embryos (Figure 6A). Similarly, although infrequent NTDs were observed among *Gldc*^*GT2/+*^ heterozygotes, they did not arise in *Mthfr* ^*−/−*^; *Gldc*^*GT2/+*^ embryos.

LC-MS/MS-based folate profiling of the *Gldc/Mthfr* compound mutant embryos at E11.5 showed significant variation between genotypes in the relative abundance of each folate (Figure 6B). This was largely driven by the *Mthfr* genotype in that the *Mthfr*^*−/−*^ embryos differed from *Mthfr*^*+/+*^ in abundance of every folate, irrespective of *Gldc* genotype (Figure 6B). Hence, the abundance of 5-methyl THF was significantly diminished in *Mthfr*-null embryos, and the relative abundance of the other folates, including formyl-THF and THF, was correspondingly increased in *Mthfr*^*−/−*^; *Gldc*^*GT2/GT2*^ compared with *Mthfr*^*+/+*^; *Gldc*^*GT2/GT2*^ embryos. Loss of function of *Gldc* caused an additional increase in dihydrofolate (DHF) and a decrease in methylene-THF in double-mutant *Mthfr*^*−/−*^; *Gldc*^*GT2/GT2*^ embryos compared with *Mthfr*^*−/−*^*; Gldc*^*+/+*^ (Figure 6B; bars marked ^∗∗^ differ from all other genotypes). In the presence of wild-type *Mthfr*, loss of function of *Gldc* conferred an increase in abundance of DHF and THF and a reduction in 5-methyl THF, as observed in *Gldc*^*GT1*^ embryos (Pai et al., 2015).

Quantification of embryonic tissue SAM showed no effect of *Gldc* or *Mthfr* genotype, whereas SAH abundance was significantly increased in the absence of *Mthfr* but not *Gldc* (Figure 6C). Hence, preventing transfer of 1C units from the folate cycle to the methionine cycle prevents NTDs in *Gldc-*deficient embryos.

We asked whether loss of Mthfr activity also modifies susceptibility to NTDs induced by pharmacological disruption of the folate cycle. Maternal treatment with methotrexate, an inhibitor of dihydrofolate reductase (Dhfr) is reported to induce NTDs (Zhao et al., 2013). However, using similar doses, we did not observe NTDs in embryos of any genotype among litters generated by intercrossing of *Mthfr*^*+/−*^ mice (Table S5). As an alternative approach, we tested the effect of maternal treatment with 5-fluorouracil, a thymidylate synthase inhibitor. Single doses of 28 or 40 mg/kg at E8.5 resulted in embryonic death (resorption) or severe retardation of growth and development, so cranial neural tube closure could not be assessed. Among 22 litters (165 embryos) treated with a lower dose of 5-fluorouracil (12.5 or 20 mg/kg), NTDs were observed at low frequency (Table S5; Figure S2). Compared with *Mthfr*^+/+^ embryos (11.6% NTDs), we noted a trend toward a lower frequency of NTDs among littermates carrying one (*Mthfr*^*+/−*^; 5.0% NTDs) or two (*Mthfr*^*−/−*^; 2.9%) null alleles (Table S5).

**Figure 7:**
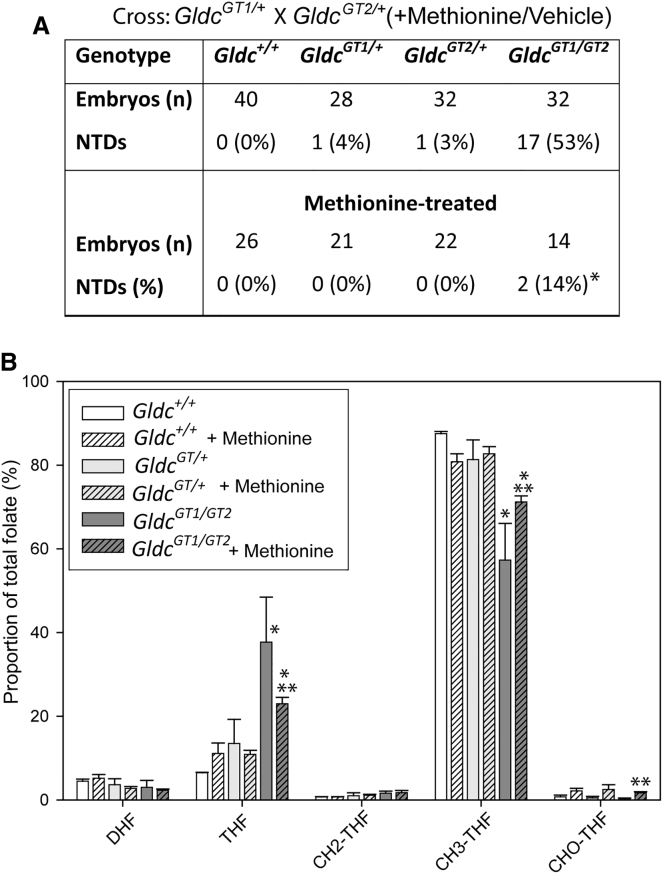
Methionine Supplementation of *Gldc*-Deficient Embryos Is Associated with Normalization of the Folate Profile and Prevention of NTDs (A) Compared with controls (n = 20 litters), maternal supplementation with methionine from E7.5 (n = 12 litters) resulted in a lower frequency of NTDs among homozygous mutant embryos (significant difference from controls; ^∗^p < 0.05, Fisher’s exact test). (B) Folate profile of embryos collected at E10.5 following maternal methionine treatment (n = 3–6 embryos per group). Significant differences in relative abundance between genotypes and/or treatment were noted for THF, 5-methyl THF, and formyl THF (p < 0.001, two-way ANOVA; ^∗^, significant difference from other genotypes within treatment group; ^∗∗^, significant effect of methionine treatment within the same genotype).

Finally, because the *Mthfr/Gldc* mice (above) were not fully congenic, we further tested whether modulation of the methionine cycle affects neural tube closure in *Gldc-*deficient embryos even though there is no apparent deficit of methionine cycle metabolites. We predicted that provision of supplemental methionine may alter partitioning of 1C units from the folate cycle, as in *Mthfr*-null embryos. Litters were generated by intercrossing of *Gldc*^*GT1/+*^ and *Gldc*^*GT2/+*^ mice and treated by maternal supplementation with methionine (70 mg/kg, n = 20 control and 12 methionine litters). Notably, we observed a significantly lower frequency of cranial NTDs among methionine-treated *Gldc*^*GT1/GT2*^ embryos compared with controls (Figure 7A). Methionine treatment appeared to normalize the folate profile of *Gldc*-deficient embryos. In control litters, *Gldc*^*GT1/GT2*^ embryos exhibit a higher relative abundance of THF and a decreased relative abundance of 5-methyl THF at E10.5 compared with *Gldc*^*+/+*^ littermates. The magnitude of these abnormalities was diminished in methionine-treated embryos (Figure 7B). Notably, the relative abundance of formyl THF was extremely low in untreated *Gldc-*deficient embryos but was increased by methionine treatment (Figure 7B). These findings are consistent with the hypothesis that supplemental methionine “spares” folate-derived 1C units that are retained in the folate cycle and support functions that are essential for neural tube closure.

## Discussion

Folate status is associated with risk of NTDs in human clinical trials and population studies (Blom et al., 2006, Copp et al., 2013, Crider et al., 2011). The link between the folate cycle and methionine cycle in FOCM has led to the hypothesis of a mechanistic sequence from disruption of folate-dependent reactions to impaired methylation and consequent failure of neural tube closure. This model is widely cited but appears to be an over-simplification. Our findings suggest that regulation of the partitioning of 1C units between mitochondrial FOCM, the folate cycle, and flux through the methionine cycle is required for neural tube closure.

Although inhibitor studies need careful interpretation, for potential non-specific effects, they provide experimental evidence that methionine cycle function is essential for neural tube closure in mice (Dunlevy et al., 2006; this study), chicks (Afman et al., 2005) and *Xenopus* (Toriyama et al., 2017). Plausible mechanisms that may underlie NTDs include diminished methylation of genomic DNA (Okano et al., 1999), proteins (Moephuli et al., 1997, Toriyama et al., 2017), or other molecules. Nevertheless, analysis of *Mthfr*-null embryos shows that generation of 5-methyl THF within the neurulation stage embryo is not essential for maintenance of SAM abundance or for neural tube closure. Moreover, concomitant loss of both maternal and embryonic Mthfr activity does not cause NTDs. These observations suggest that, despite a significant reduction in the SAM/SAH ratio, the availability of SAM for methylation is adequate to meet demand during neurulation (despite post-natal abnormalities). Similarly, dietary folate deficiency causes a reduction in the SAM/SAH ratio without causing NTDs in wild-type embryos (Burren et al., 2008). These observations suggest that maternal methionine intake and/or use of choline and betaine as sources of 1C groups for methylation of homocysteine by betaine-homocysteine methyltransferase (Bhmt) is sufficient to meet the demand for methionine in the neurulating embryo (for production of SAM and for protein synthesis). In fact, both may play a role. We found that SAH is elevated in *Mthfr*-null embryos (suggesting a limit on homocysteine remethylation by Bhmt), whereas SAM is diminished in *Mthfr*-null tissues at 3 weeks of age (suggesting that dietary methionine is insufficient to meet the demand, at least post-natally). Interestingly, lowering of SAM levels in mouse blastocysts requires concomitant suppression of both the folate cycle and Bhmt expression (Zhang et al., 2015). The reduction in plasma betaine levels in *Mthfr*-null adult mice suggests increased used of the choline-betaine-dependent pathway for methylation reactions (Schwahn et al., 2003).

How do findings in mice relate to human NTDs? Although evaluation of the effect of maternal folate status on DNA methylation within the neurulation stage human embryo is practically difficult, associations have been observed in analysis of cord blood (Joubert et al., 2016). Notably however, the majority of reported studies show a negative association, arguing against a generalized methylation-promoting effect of folic acid. Whether specific genomic loci or other targets, such as proteins or RNA, are targeted remains to be determined. Human studies of association of blood choline levels with NTDs have been inconsistent, suggesting that, if an effect is present, then it is unlikely to be strong (Mills et al., 2014). Interestingly however, association with NTDs has been noted for polymorphisms in *PEMT*, encoding phosphatidylethanolamine N-methyltransferase, which mediates phosphatidylcholine biosynthesis (Zhang et al., 2006, Mills et al., 2014). Moreover, use of choline as a methyl donor in production of phosphatidylcholine appears to be favored in individuals carrying the rs1801133 polymorphism in *MTHFR* (Yan et al., 2011). It is possible that the potential association of choline status with NTDs needs to be analyzed in the context of genotype for both *MTHFR* and *PEMT*.

Although transfer of 1C units from the folate cycle to the methionine cycle in embryonic or maternal FOCM appears to be dispensable for neural tube closure in mice, we find that generation of 1C units in mitochondrial FOCM is essential. Hence, NTDs caused by loss of function of the GCS result from lack of 1C transfer to THF, with consequent suppression of 5,10-methylene THF synthesis and subsequent 1C-dependent reactions. This model is consistent with prevention of NTDs by formate, which occurs even though elevated glycine is still present in formate-treated embryos (Pai et al., 2015). The consequence of GCS disruption in the embryo therefore differs from effects of elevated glycine in some cancer cell lines, where elevated glycine reverses Shmt-mediated reactions and inhibits proliferation (Labuschagne et al., 2014) or even has toxic effects (Kim et al., 2015).

Isotope labeling demonstrated that glycine contributes to *de novo* purine synthesis both by direct incorporation into the purine ring and via donation of 1C groups via 10-formyl THF. The dependence on two 10-formyl THF units may confer particular sensitivity of purine biosynthesis to impaired glycine cleavage compared with thymidylate biosynthesis, which requires only one 1C group.

Having established that GCS-derived 1C units contribute to cytoplasmic FOCM in the embryo, we found no effect of *Gldc* deficiency on the abundance of SAM, SAH, or the SAM/SAH ratio, suggesting that *Gldc-*related NTDS are unlikely to result from impaired methionine cycle activity. This conclusion is consistent with the lack of NTDs in *Mthfr*^−/−^ embryos even though SAH concentration is elevated, although SAM-mediated methylation is limiting for neural tube closure based on the evidence from use of methylation cycle inhibitors. On the other hand, the striking effect of Mthfr loss of function in preventing NTDs in *Gldc*-null embryos suggests that retention of 1C units within the folate cycle, at the expense of the methionine cycle, ameliorates the diminished supply from glycine cleavage. These findings suggest a crucial requirement for the folate cycle and/or mitochondrial FOCM in neural tube closure; key outputs likely include nucleotide biosynthesis as well as potentially other outputs of these cycles. The protective effect of Mthfr ablation is also consistent with the protective effect of methionine in *Gldc*-null embryos (this study) and in *Amt*^*−/−*^ embryos (Narisawa et al., 2012). We hypothesize that addition of methionine spares 1C units from transfer to the methionine cycle. In both Mthfr-null and methionine-treated embryos, prevention of NTDs correlates with an increase in relative abundance of 10-formyl THF.

The protective effect of Mthfr loss of function in a mouse mutant with NTDs could be considered counterintuitive in the context that the rs1801133 (C677T) polymorphism is an established risk factor for NTD (Botto and Yang, 2000, Blom et al., 2006, Greene et al., 2009). However, this polymorphism is associated with lower plasma and red blood cell folate levels (Crider et al., 2011, Tsang et al., 2015). Our findings are consistent with the hypothesis that the NTD risk association of *MTHFR* is mediated through the effect on blood folate status (Stover et al., 2015) rather than an effect on enzymatic activity leading to diminished methylation.

## Experimental Procedures

### Mice

*Mthfr*-null mice were described previously (Chen et al., 2001). To generate *Mthfr*-null dams for experimental matings, early lethality was prevented by maternal supplementation with betaine until weaning (Schwahn et al., 2004). *Gldc*-deficient mice (denoted *Gldc*^*GT1*^) were described previously (Pai et al., 2015). An additional line of *Gldc*-deficient mice (denoted *Gldc*^*GT2*^) was generated using an embryonic stem cell line obtained from the North American Conditional Mouse Mutagenesis (NorCOMM) project. Animal studies were carried out under the regulations of the Animals (Scientific Procedures) Act 1986 of the United Kingdom Government and in accordance with the guidance issued by the Medical Research Council, United Kingdom in *Responsibility in the Use of Animals for Medical Research* (July 1993).

Litters were generated by timed matings in which mice (used from 7 weeks of age) were paired overnight, and the day of finding a copulation plug was designated E0.5. Whole-embryo culture was performed as described previously (Dunlevy et al., 2006, Pryor et al., 2012). Mice and embryos were genotyped by PCR of genomic DNA.

### Quantification of FOCM Intermediates by Mass Spectrometry

Analysis of multiple folates was performed by ultra-performance LC (UPLC)-MS/MS as described previously (Pai et al., 2015). Folates were measured by multiple reaction monitoring (MRM) with optimized cone voltage and collision energy for precursor and product ions as described previously (Leung et al., 2013, Pai et al., 2015). SAM and SAH were quantified by LC-MS/MS as described previously (Burren et al., 2006) with minor modifications. Cytosine 5-methylation of DNA was analyzed by LC mass spectrometry using a method that is insensitive to co-occurring RNA methylation (Capuano et al., 2014). See the Supplemental Experimental Procedures for details.

### Stable Isotope Tracing of [1,2-^13^C] Glycine

Embryo extracts were analyzed by LC-MS as described previously (Chen et al., 2012, Ismailoglu et al., 2014). We applied an in-house untargeted stable isotope tracing (USIT) workflow in which labeled metabolites were identified on the basis of differential abundance in embryos cultured in [1,2-^13^C] glycine compared with [1,2-^12^C] glycine-supplemented serum. Selected metabolites were identified on the basis of previously curated isotopologues.

### Statistical Analysis

Quantitative data (e.g., metabolite abundance) was analyzed by t test (two groups) or ANOVA (three or more groups) with Holm-Sidak pairwise comparison for *post hoc* analysis. Prior to application of these methods, data were checked for normal distribution. Analysis of the frequency of *Gldc* mutant embryos that exhibited NTDs in different treatment groups or by *Mthfr* genotype was performed by Fisher’s exact test. Statistical analysis was performed using Sigmastat (v3.5, Systat Software).

## Author Contributions

Conceptualization, N.D.E.G., K.-Y.L., and A.J.C.; Methodology, Q.C., S.S.G., K.-Y.L., and M.R.; Formal Analysis, N.D.E.G. and K.-Y.L.; Investigation, K.-Y.L., Y.J.P., Q.C., C.S., E.C., S.S., D.S., and N.D.E.G.; Writing – Original Draft, N.D.E.G; Writing – Review & Editing, N.D.E.G., K.-Y.L., and A.J.C.; Visualization, N.D.E.G., K.-Y.L., and Q.C.; Supervision, N.D.E.G., M.R., and S.S.G.

## Supplemental Information

Document S1. Supplemental Experimental Procedures, Figures S1 and S2, and Tables S1–S5

Document S2. Article plus Supplemental Information

## References

[bib1] Afman L.A., Blom H.J., Drittij M.J., Brouns M.R., van Straaten H.W. (2005). Inhibition of transmethylation disturbs neurulation in chick embryos. Brain Res. Dev. Brain Res..

[bib2] Alix J.-H. (1982). Molecular aspects of the in vivo and in vitro effects of ethionine, an analog of methionine. Microbiol. Rev..

[bib3] Beaudin A.E., Abarinov E.V., Noden D.M., Perry C.A., Chu S., Stabler S.P., Allen R.H., Stover P.J. (2011). Shmt1 and de novo thymidylate biosynthesis underlie folate-responsive neural tube defects in mice. Am. J. Clin. Nutr..

[bib4] Blom H.J., Shaw G.M., den Heijer M., Finnell R.H. (2006). Neural tube defects and folate: case far from closed. Nat. Rev. Neurosci..

[bib5] Botto L.D., Yang Q. (2000). 5,10-Methylenetetrahydrofolate reductase gene variants and congenital anomalies: a HuGE review. Am. J. Epidemiol..

[bib6] Brosnan M.E., MacMillan L., Stevens J.R., Brosnan J.T. (2015). Division of labour: how does folate metabolism partition between one-carbon metabolism and amino acid oxidation?. Biochem. J..

[bib7] Burren K.A., Mills K., Copp A.J., Greene N.D.E. (2006). Quantitative analysis of s-adenosylmethionine and s-adenosylhomocysteine in neurulation-stage mouse embryos by liquid chromatography tandem mass spectrometry. J. Chromatogr. B Analyt. Technol. Biomed. Life Sci..

[bib8] Burren K.A., Savery D., Massa V., Kok R.M., Scott J.M., Blom H.J., Copp A.J., Greene N.D.E. (2008). Gene-environment interactions in the causation of neural tube defects: folate deficiency increases susceptibility conferred by loss of *Pax3* function. Hum. Mol. Genet..

[bib9] Burren K.A., Scott J.M., Copp A.J., Greene N.D. (2010). The genetic background of the curly tail strain confers susceptibility to folate-deficiency-induced exencephaly. Birth Defects Res. A Clin. Mol. Teratol..

[bib10] Capuano F., Mülleder M., Kok R., Blom H.J., Ralser M. (2014). Cytosine DNA methylation is found in Drosophila melanogaster but absent in Saccharomyces cerevisiae, Schizosaccharomyces pombe, and other yeast species. Anal. Chem..

[bib11] Caudill M.A., Wang J.C., Melnyk S., Pogribny I.P., Jernigan S., Collins M.D., Santos-Guzman J., Swendseid M.E., Cogger E.A., James S.J. (2001). Intracellular S-adenosylhomocysteine concentrations predict global DNA hypomethylation in tissues of methyl-deficient cystathionine beta-synthase heterozygous mice. J. Nutr..

[bib12] Chen Z., Karaplis A.C., Ackerman S.L., Pogribny I.P., Melnyk S., Lussier-Cacan S., Chen M.F., Pai A., John S.W., Smith R.S. (2001). Mice deficient in methylenetetrahydrofolate reductase exhibit hyperhomocysteinemia and decreased methylation capacity, with neuropathology and aortic lipid deposition. Hum. Mol. Genet..

[bib13] Chen Q., Park H.C., Goligorsky M.S., Chander P., Fischer S.M., Gross S.S. (2012). Untargeted plasma metabolite profiling reveals the broad systemic consequences of xanthine oxidoreductase inactivation in mice. PLoS ONE.

[bib14] Copp A.J., Stanier P., Greene N.D. (2013). Neural tube defects: recent advances, unsolved questions, and controversies. Lancet Neurol..

[bib15] Crider K.S., Bailey L.B., Berry R.J. (2011). Folic acid food fortification-its history, effect, concerns, and future directions. Nutrients.

[bib16] Davis S.R., Stacpoole P.W., Williamson J., Kick L.S., Quinlivan E.P., Coats B.S., Shane B., Bailey L.B., Gregory J.F. (2004). Tracer-derived total and folate-dependent homocysteine remethylation and synthesis rates in humans indicate that serine is the main one-carbon donor. Am. J. Physiol. Endocrinol. Metab..

[bib17] De Castro S.C., Leung K.Y., Savery D., Burren K., Rozen R., Copp A.J., Greene N.D.E. (2010). Neural tube defects induced by folate deficiency in mutant curly tail (Grhl3) embryos are associated with alteration in folate one-carbon metabolism but are unlikely to result from diminished methylation. Birth Defects Res. A Clin. Mol. Teratol..

[bib18] Ducker G.S., Rabinowitz J.D. (2017). One-Carbon Metabolism in Health and Disease. Cell Metab..

[bib19] Dunlevy L.P.E., Burren K.A., Mills K., Chitty L.S., Copp A.J., Greene N.D.E. (2006). Integrity of the methylation cycle is essential for mammalian neural tube closure. Birth Defects Res. A Clin. Mol. Teratol..

[bib20] Fan J., Ye J., Kamphorst J.J., Shlomi T., Thompson C.B., Rabinowitz J.D. (2014). Quantitative flux analysis reveals folate-dependent NADPH production. Nature.

[bib21] Fleming A., Copp A.J. (1998). Embryonic folate metabolism and mouse neural tube defects. Science.

[bib22] Ghandour H., Chen Z., Selhub J., Rozen R. (2004). Mice deficient in methylenetetrahydrofolate reductase exhibit tissue-specific distribution of folates. J. Nutr..

[bib23] Greene N.D.E., Stanier P., Copp A.J. (2009). Genetics of human neural tube defects. Hum. Mol. Genet..

[bib24] Heid M.K., Bills N.D., Hinrichs S.H., Clifford A.J. (1992). Folate deficiency alone does not produce neural tube defects in mice. J. Nutr..

[bib25] Herbig K., Chiang E.P., Lee L.R., Hills J., Shane B., Stover P.J. (2002). Cytoplasmic serine hydroxymethyltransferase mediates competition between folate-dependent deoxyribonucleotide and S-adenosylmethionine biosyntheses. J. Biol. Chem..

[bib26] Ismailoglu I., Chen Q., Popowski M., Yang L., Gross S.S., Brivanlou A.H. (2014). Huntingtin protein is essential for mitochondrial metabolism, bioenergetics and structure in murine embryonic stem cells. Dev. Biol..

[bib27] Jadavji N.M., Deng L., Malysheva O., Caudill M.A., Rozen R. (2015). MTHFR deficiency or reduced intake of folate or choline in pregnant mice results in impaired short-term memory and increased apoptosis in the hippocampus of wild-type offspring. Neuroscience.

[bib28] Jain M., Nilsson R., Sharma S., Madhusudhan N., Kitami T., Souza A.L., Kafri R., Kirschner M.W., Clish C.B., Mootha V.K. (2012). Metabolite profiling identifies a key role for glycine in rapid cancer cell proliferation. Science.

[bib29] Joubert B.R., den Dekker H.T., Felix J.F., Bohlin J., Ligthart S., Beckett E., Tiemeier H., van Meurs J.B., Uitterlinden A.G., Hofman A. (2016). Maternal plasma folate impacts differential DNA methylation in an epigenome-wide meta-analysis of newborns. Nat. Commun..

[bib30] Kalhan S.C., Hanson R.W. (2012). Resurgence of serine: an often neglected but indispensable amino Acid. J. Biol. Chem..

[bib31] Kim D., Fiske B.P., Birsoy K., Freinkman E., Kami K., Possemato R.L., Chudnovsky Y., Pacold M.E., Chen W.W., Cantor J.R. (2015). SHMT2 drives glioma cell survival in ischaemia but imposes a dependence on glycine clearance. Nature.

[bib32] Krupenko N.I., Dubard M.E., Strickland K.C., Moxley K.M., Oleinik N.V., Krupenko S.A. (2010). ALDH1L2 is the mitochondrial homolog of 10-formyltetrahydrofolate dehydrogenase. J. Biol. Chem..

[bib33] Labuschagne C.F., van den Broek N.J., Mackay G.M., Vousden K.H., Maddocks O.D. (2014). Serine, but not glycine, supports one-carbon metabolism and proliferation of cancer cells. Cell Rep..

[bib34] Lamers Y., Williamson J., Gilbert L.R., Stacpoole P.W., Gregory J.F. (2007). Glycine turnover and decarboxylation rate quantified in healthy men and women using primed, constant infusions of [1,2-(13)C2]glycine and [(2)H3]leucine. J. Nutr..

[bib35] Lamers Y., Williamson J., Theriaque D.W., Shuster J.J., Gilbert L.R., Keeling C., Stacpoole P.W., Gregory J.F. (2009). Production of 1-carbon units from glycine is extensive in healthy men and women. J. Nutr..

[bib36] Lawrance A.K., Racine J., Deng L., Wang X., Lachapelle P., Rozen R. (2011). Complete deficiency of methylenetetrahydrofolate reductase in mice is associated with impaired retinal function and variable mortality, hematological profiles, and reproductive outcomes. J. Inherit. Metab. Dis..

[bib37] Leung K.Y., De Castro S.C., Cabreiro F., Gustavsson P., Copp A.J., Greene N.D. (2013). Folate metabolite profiling of different cell types and embryos suggests variation in folate one-carbon metabolism, including developmental changes in human embryonic brain. Mol. Cell. Biochem..

[bib38] Locasale J.W. (2013). Serine, glycine and one-carbon units: cancer metabolism in full circle. Nat. Rev. Cancer.

[bib39] MacFarlane A.J., Anderson D.D., Flodby P., Perry C.A., Allen R.H., Stabler S.P., Stover P.J. (2011). Nuclear localization of de novo thymidylate biosynthesis pathway is required to prevent uracil accumulation in DNA. J. Biol. Chem..

[bib40] Mills J.L., Fan R., Brody L.C., Liu A., Ueland P.M., Wang Y., Kirke P.N., Shane B., Molloy A.M. (2014). Maternal choline concentrations during pregnancy and choline-related genetic variants as risk factors for neural tube defects. Am. J. Clin. Nutr..

[bib41] Moephuli S.R., Klein N.W., Baldwin M.T., Krider H.M. (1997). Effects of methionine on the cytoplasmic distribution of actin and tubulin during neural tube closure in rat embryos. Proc. Natl. Acad. Sci. USA.

[bib42] Momb J., Lewandowski J.P., Bryant J.D., Fitch R., Surman D.R., Vokes S.A., Appling D.R. (2013). Deletion of Mthfd1l causes embryonic lethality and neural tube and craniofacial defects in mice. Proc. Natl. Acad. Sci. USA.

[bib43] Narisawa A., Komatsuzaki S., Kikuchi A., Niihori T., Aoki Y., Fujiwara K., Tanemura M., Hata A., Suzuki Y., Relton C.L. (2012). Mutations in genes encoding the glycine cleavage system predispose to neural tube defects in mice and humans. Hum. Mol. Genet..

[bib44] Okano M., Bell D.W., Haber D.A., Li E. (1999). DNA methyltransferases Dnmt3a and Dnmt3b are essential for de novo methylation and mammalian development. Cell.

[bib45] Pai Y.J., Leung K.Y., Savery D., Hutchin T., Prunty H., Heales S., Brosnan M.E., Brosnan J.T., Copp A.J., Greene N.D. (2015). Glycine decarboxylase deficiency causes neural tube defects and features of non-ketotic hyperglycinemia in mice. Nat. Commun..

[bib46] Pryor S.E., Massa V., Savery D., Greene N.D.E., Copp A.J. (2012). Convergent extension analysis in mouse whole embryo culture. Methods Mol. Biol..

[bib47] Reed M.C., Nijhout H.F., Neuhouser M.L., Gregory J.F., Shane B., James S.J., Boynton A., Ulrich C.M. (2006). A mathematical model gives insights into nutritional and genetic aspects of folate-mediated one-carbon metabolism. J. Nutr..

[bib48] Schwahn B.C., Chen Z., Laryea M.D., Wendel U., Lussier-Cacan S., Genest J., Mar M.H., Zeisel S.H., Castro C., Garrow T., Rozen R. (2003). Homocysteine-betaine interactions in a murine model of 5,10-methylenetetrahydrofolate reductase deficiency. FASEB J..

[bib49] Schwahn B.C., Laryea M.D., Chen Z., Melnyk S., Pogribny I., Garrow T., James S.J., Rozen R. (2004). Betaine rescue of an animal model with methylenetetrahydrofolate reductase deficiency. Biochem. J..

[bib50] Scott J.M. (1999). Folate and vitamin B12. Proc. Nutr. Soc..

[bib51] Shah R.H., Northrup H., Hixson J.E., Morrison A.C., Au K.S. (2016). Genetic association of the glycine cleavage system genes and myelomeningocele. Birth Defects Res. A Clin. Mol. Teratol..

[bib52] Spiegelstein O., Mitchell L.E., Merriweather M.Y., Wicker N.J., Zhang Q., Lammer E.J., Finnell R.H. (2004). Embryonic development of folate binding protein-1 (Folbp1) knockout mice: Effects of the chemical form, dose, and timing of maternal folate supplementation. Dev. Dyn..

[bib53] Stover P.J., MacFarlane A.J., Field M.S. (2015). Bringing clarity to the role of MTHFR variants in neural tube defect prevention. Am. J. Clin. Nutr..

[bib54] Tibbetts A.S., Appling D.R. (2010). Compartmentalization of Mammalian folate-mediated one-carbon metabolism. Annu. Rev. Nutr..

[bib55] Toriyama M., Toriyama M., Wallingford J.B., Finnell R.H. (2017). Folate-dependent methylation of septins governs ciliogenesis during neural tube closure. FASEB J..

[bib56] Tsang B.L., Devine O.J., Cordero A.M., Marchetta C.M., Mulinare J., Mersereau P., Guo J., Qi Y.P., Berry R.J., Rosenthal J. (2015). Assessing the association between the methylenetetrahydrofolate reductase (MTHFR) 677C>T polymorphism and blood folate concentrations: a systematic review and meta-analysis of trials and observational studies. Am. J. Clin. Nutr..

[bib57] Yan J., Wang W., Gregory J.F., Malysheva O., Brenna J.T., Stabler S.P., Allen R.H., Caudill M.A. (2011). MTHFR C677T genotype influences the isotopic enrichment of one-carbon metabolites in folate-compromised men consuming d9-choline. Am. J. Clin. Nutr..

[bib58] Yang M., Vousden K.H. (2016). Serine and one-carbon metabolism in cancer. Nat. Rev. Cancer.

[bib59] Zhang J., Zhu H., Yang W., Shaw G.M., Lammer E.J., Finnell R.H. (2006). Phosphatidylethanolamine N-methyltransferase (PEMT) gene polymorphisms and risk of spina bifida. Am. J. Med. Genet. A..

[bib60] Zhang B., Denomme M.M., White C.R., Leung K.Y., Lee M.B., Greene N.D., Mann M.R., Trasler J.M., Baltz J.M. (2015). Both the folate cycle and betaine-homocysteine methyltransferase contribute methyl groups for DNA methylation in mouse blastocysts. FASEB J..

[bib61] Zhao J., Guan T., Wang J., Xiang Q., Wang M., Wang X., Guan Z., Xie Q., Niu B., Zhang T. (2013). Influence of the antifolate drug Methotrexate on the development of murine neural tube defects and genomic instability. J. Appl. Toxicol..

