# Supplementary material for: Partitioning of One-Carbon Units in Folate and Methionine Metabolism Is Essential for Neural Tube Closure
Source: Cell Rep. 2017 Nov 14;21(7):1795–808. doi: 10.1016/j.celrep.2017.10.072 (PMC5699646; doi:10.1016/j.celrep.2017.10.072)
Supplement: Document S1. Supplemental Experimental Procedures, Figures S1 and S2, and Tables S1–S5 [file mmc1.pdf]

Cell Reports, Volume *21*

Supplemental Information

Partitioning of One-Carbon Units in Folate and Methionine Metabolism Is Essential for Neural Tube Closure

Kit-Yi Leung, Yun Jin Pai, Qiuying Chen, Chloe Santos, Enrica Calvani, Sonia Sudiwala, Dawn Savery, Markus Ralser, Steven S. Gross, Andrew J. Copp, and Nicholas D.E. Greene

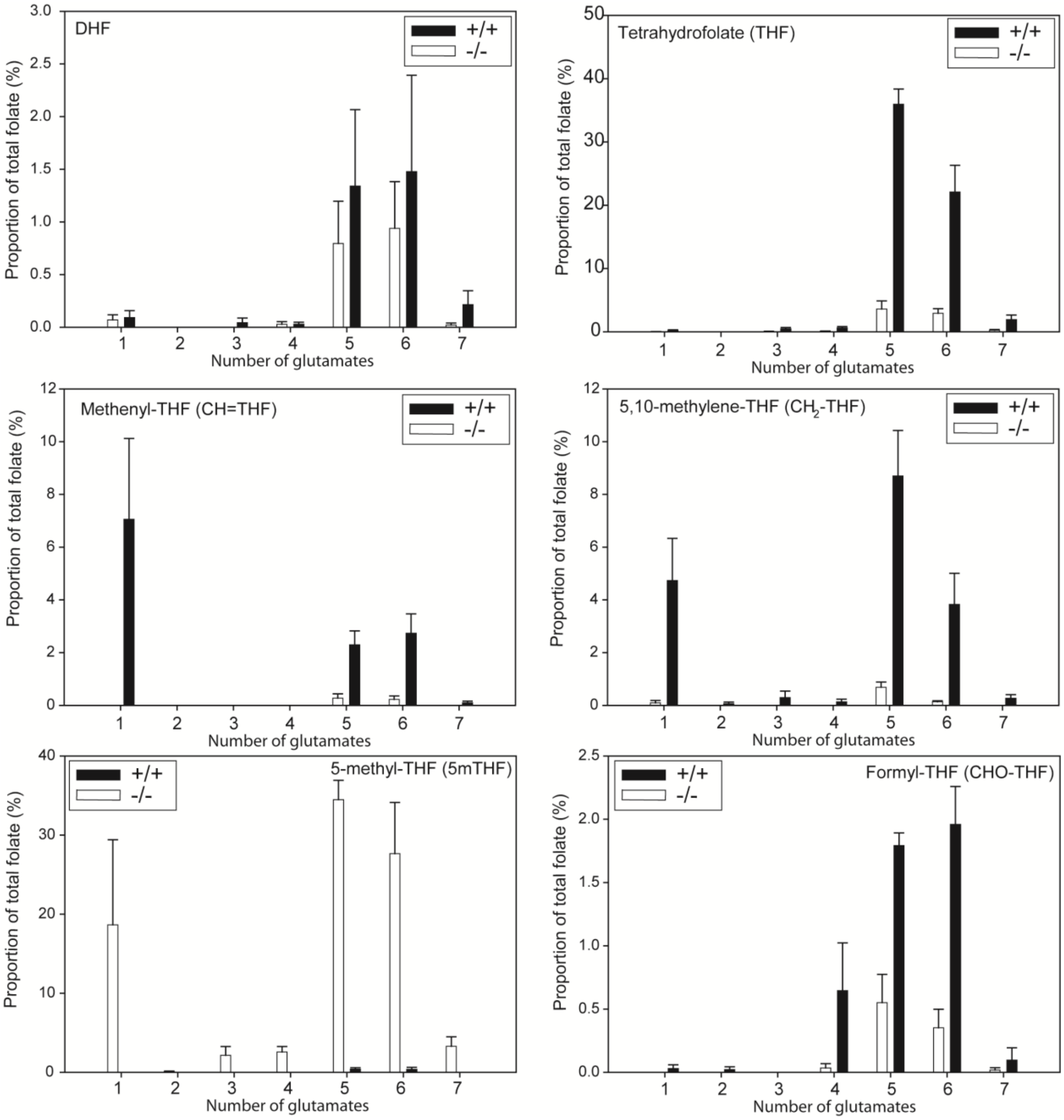

Figure S1. Folate profile of *Mthfr* wild-type and homozygous null embryos at E10.5 (Related to Figure 1). Graphs show relative proportions (expressed as % of total folate) of mono- and polyglutamated forms (n = 4 *Mthfr*$^{+/+}$ and 5 *Mthfr*$^{-/-}$).

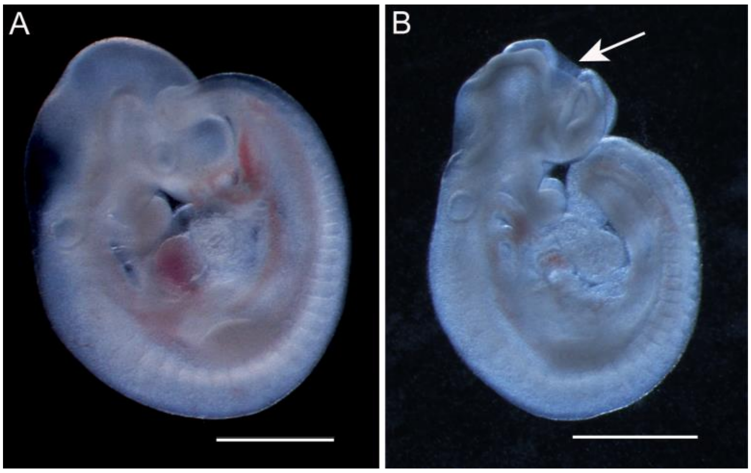

Figure S2. ***Mthfr$^{-/-}$*** **embryos at E10.5 following maternal treatment with 5-fluorouracil (20 mg/kg) at E8.5 (Related to Figure 6).** At this stage (~26 somites), cranial neural tube closure should be complete (A). NTDs are detectable as open cranial neural folds (arrow in B). Scale bar represents 1 mm.

	NTDs/embryos			
Experimental cross	**Total embryos**	***Mthfr*$^{+/+}$**	***Mthfr*$^{+/-}$**	***Mthfr*$^{-/-}$**
Mthfr$^{+/-}$ x *Mthfr*$^{+/-}$	0/205	0/48	0/111	0/46
Mthfr$^{+/-}$ x *Mthfr*$^{-/-}$	0/17	-	0/10	0/7

Supplementary Table S1. Litters collected from intercross of *Mthfr*$^{+/-}$ males with *Mthfr*$^{+/-}$ or *Mthfr*$^{-/-}$ females (Related to Figure 1). NTDs were not observed among embryos of any genotype (collected at E10.5-12.5).

**Genotype**	**No. embryos (n)**	**SAM (nmol/mg/protein)**	**SAH (nmol/mg protein)**	**SAM/SAH ratio**
***Mthfr*$^{+/-}$**	6	417.2 ± 88.8	11.2 ± 1.8	35.8 ± 3.1
***Mthfr*$^{-/-}$**	4	419.2 ± 38.9	90.1 ± 17.7*	4.97 ± 0.63*

Supplementary Table S2. Methylation cycle intermediates among offspring of *Mthfr* null dams (Related to Figure 2). Litters from intercrosses of *Mthfr*$^{-/-}$ females with *Mthfr*$^{+/-}$ males were analysed at E10.5. SAH abundance and SAM/SAH ratio significantly differ between genotypes (* $p<0.001$; t-test). Values are mean ± SEM.

Genotype	$Gldc^{+/+}$	$Gldc^{GT1/+}$	$Gldc^{GT1/GT1}$
**Embryos (n)**	152	301	167
**NTDs (%)**	0 (0%)	2 (1%)	31 (19%)
	$Gldc^{+/+}$	$Gldc^{GT2/+}$	$Gldc^{GT2/GT2}$
**Embryos (n)**	69	177	62
**NTDs (%)**	0 (0%)	10 (6%)	35 (57%)

Supplementary Table S3. Incidence of NTDs among Gldc-deficient embryos (Related to Figure 4). The frequency of genotypes did not differ from the predicted Mendelian ratio. Data for NTDs are from 88 $Gldc^{GT1/+}$ intercross litters and 38 $Gldc^{GT2/+}$ intercross litters (data for $Gldc^{GT2}$ are reproduced from Fig. 4 for ease of comparison of the two strains).

	$Gldc^{+/+}$	$Gldc^{GT1/+}$	$Gldc^{GT1/GT1}$
Serine (nmol/mg protein)	17.7 ± 1.86	19.5 ± 1.75	17.4 ± 3.70

Supplementary Table S4 (Related to Figure 5). Embryo tissue serine concentration. No significant differences were detected between genotypes among embryos at E11.5 (n = 6-7 samples per genotype).

			NTDs/No. embryos			*Growth retarded*	*Resorptions*
	**Litters**	**Embryos**	***Mthfr*** $^{+/+}$	***Mthfr*** $^{+/-}$	***Mthfr*** $^{-/-}$		**Not genotyped**
**Controls**	29	205	0/48	0/111	0/46	0	14
**Methotrexate**							
4.5 mg/kg	4	24	0/4	0/12	0/8	0	8
6.0 mg/kg	5	18	0/6	0/9	0/3	0	12
**5-Fluorouracil**							
12.5 mg/kg	2	16	1/5 (20%)	0/9 (0%)	0/2	1 (6.3%)	0
20 mg/kg	20	149	4/38 (10.5%)	4/71 (5.6%)	1/33 (3.0%)	11 (7.4%)	15
28 mg/kg	2	9	-	-	-	9 (100%)	1
40/mg/kg	2	6	-	-	-	6 (100%	8

Supplementary Table S5. 5-Fluorouracil and methotrexate treatment of *Mthfr* litters (Related to Figure 6). Treatment with methotrexate was performed by intra-peritoneal injection at E7.5 (4.5 mg/kg) or E8.5 (6 mg/kg) and embryos analysed at E10.5. Dams were treated with 5-fluorouracil by intraperitoneal injection at E8.5 and embryos were analysed at E10.5. Embryos that were severely growth retarded (fewer than 15 somites and/or incomplete turning) were excluded from analysis of neural tube closure. There was a trend towards lower rate of NTDs in with number of *Mthfr* null alleles (not statistically significant).

Supplementary Experimental Procedures

Mice

Mthfr null mice were described previously (Chen et al., 2001). In order to generate *Mthfr* null dams for experimental matings, early lethality was prevented by maternal supplementation with betaine until weaning (Schwahn et al., 2004). Generation of *Gldc*-deficient mice (denoted $Gldc^{GT1}$) carrying a gene-trap construct in intron 2 of *Gldc* was described previously (Pai et al., 2015). An additional line of *Gldc*-deficient mice (denoted $Gldc^{GT2}$) was generated using an embryonic stem cell line (clone CMHD-GT_519C8) obtained from the North American Conditional Mouse Mutagenesis (NorCOMM) project. Chimeric mice were generated by blastocyst injection of ES cells (carried out in the Embryonic Stem Cell Facility, UCL Institute of Child Health). Mice were crossed with wild-type 129/Sv mice to confirm germ-line transmission and then back-crossed onto a C57BL/6 background to establish a heterozygous colony for generation of experimental litters. *Mthfr/Gldc* interaction studies were performed on a principally $Gldc^{GT2}$ strain (but not fully isogenic) genetic background.

Mice were used for experimental matings from six weeks of age. Mice were maintained ona standard breeder diet containing 0.5% methionine (Teklad). Litters were generated by timed matings in which mice were paired overnight and the day of finding a copulation plug was designated embryonic day 0.5 (E0.5). Pregnant females were killed by cervical dislocation at various stages from E8.5-12.5. The uterus was removed and transferred to Dulbecco's Modified Eagles Medium (DMEM; Invitrogen). For biochemical analysis tissue was rinsed in phosphate buffered saline (PBS), immediately frozen on dry ice and stored at -80°C.

Methionine, 5-fluorouracil or methotrexate were administered by intra-peritoneal injection of the pregnant dam. Animal studies were carried out under regulations of the Animals (Scientific Procedures) Act 1986 of the UK Government, and in accordance with the guidance issued by the Medical Research Council, UK in *Responsibility in the Use of Animals for Medical Research* (July 1993).

Genotyping

Mice were genotyped by PCR of genomic DNA prepared from yolk sacs as described previously (Pai et al., 2015) (Chen et al., 2001). The *Gldc*^{GT2} gene-trap construct was localised to intron 19 by PCR and sequencing of genomic DNA spanning the intron-construct junctions. *Gldc*^{GT2} mice were genotyped by PCR using primer pairs that amplify the wild-type allele (5’ TACAGTCTGTGAACGGAGTCC and 5’ TCACTTTCTGAAGGGTTGGAGAGG) or mutant allele (5’-GCGAGGAGCTGTTCACCGGG and 5’-ACCTCGGCGCGGGTCTTGTA).

Mouse embryo culture

Embryos were explanted at E8.5 or E9.5 and cultured with the yolk sac intact in rat serum for 24hr as described previously (Cockroft, 1990; Pryor et al., 2012). The cultures were gassed according to stage with 5% O_2, 5% CO_2 and 90% N_2 or 20% O_2, 5% CO_2 and 75% N_2 and 40% O_2, 5% CO_2, 55% N_2 (Pryor et al., 2012). Treatments were administered as 0.1% v/v additions from stock solutions of ethionine or cycloleucine to final concentrations of 5 mM and 15 mM, respectively (Dunlevy et al., 2006). Controls were treated with the same volume of vehicle only. For stable isotope tracing embryos were cultured with 2 mM [1,2-^{13}C] glycine or [1,2-^{12}C] glycine for 24 hr, rinsed twice in PBS and immediately frozen on dry ice.

Quantification of FOCM intermediates by mass spectrometry

Folates: Analysis of multiple folates was performed by UPLC-MS/MS as described previously (Pai et al., 2015; Leung et al., 2013; Cabreiro et al., 2013). Sample buffer containing 20mM ammonium acetate, 0.1% ascorbic acid, 0.1% citric acid and 100mM DTT at pH7 was added to frozen embryo, liver and brain tissue samples. Buffer was selected on the basis of sensitivity of MS response and stability of folates (Pai et al., 2015): at pH7 some abiotic conversion of CH_2-THF to THF occurs (maximal 20% in a mouse embryo/tissue matrix) but sensitivity is significantly greater than at pH 10 (which stabilises CH_2-THF).

Sample suspensions were sonicated for 10 seconds using a hand-held sonicator at 40% amplitude and 60% for liver. Protein was removed by precipitation with addition of 2 sample volume of acetonitrile, mixing for two minutes and centrifugation for 15 minutes at 12,000 x g and 4°C. Supernatants were transferred to fresh tubes, lyophilised and stored at -80°C prior to analysis.

Lyophilised samples were resuspended in 30µl sample buffer (as above) and centrifuged for 5 minutes at 12,000 x g at 4oC. Supernatants were transferred to glass sample vials for UPLC-MS/MS analysis. Metabolites were resolved by reversed-phase chromatography using Acquity UPLC BEH C18 column (50mm x 2.1mm; 1.7µm bead size , Waters Corporation, UK). Solvents for UPLC were: Buffer A, 5% methanol, 95% Milli-Q water and 5mM dimethylhexylamine at pH 8.0; Buffer B, 100% methanol, 5mM dimethylhexylamine. The column was equilibrated with 95% Buffer A: 5% Buffer B. The sample injection volume was 25 µl. The UPLC protocol consisted of 95% Buffer A: 5% Buffer B for 1 min, followed by a gradient of 5-60% Buffer B over 9 min and then 100% Buffer B for 6 min before re-

equilibration for 4 min. The metabolites were eluted at a flow rate of 200 nl/min. The UPLC was coupled to a XEVO-TQS mass spectrometer (Waters Corporation, UK) operating in negative-ion mode using the following settings: capillary 2.5kV, source temperature 150°C, desolvation temperature 600°C, cone gas flow rate 150 L/h and desolvation gas flow rate 1200 L/h. Folates were measured by multiple reaction monitoring (MRM) with optimised cone voltage and collision energy for precursor and product ions as described (Leung et al., 2013; Cabreiro et al., 2013).

S-Adenosylmethionine and S-adenosylhomocysteine: SAM and SAH were quantified by LC-MS/MS as described previously (Burren et al., 2006), with minor modifications to run on UPLC coupled to a XEVO-TQS mass spectrometer.

Analysis of DNA methylation

Cytosine 5-methylation of DNA was analysed by liquid chromatography mass spectrometry using a method that is insensitive to co-occurring RNA methylation, as described previously (Capuano et al., 2014). In brief, DNA was purified, digested with DNA Degradase plus (Zymo Research) and analysed on a Triple Quadrupole mass spectrometer (Agilent 6470) operating in selective reaction monitoring mode after high-pressure chromatographic separation.

Isotope tracing

Metabolite extraction and LC-MS: Embryo samples were washed twice with cold PBS, followed by bead-beating in 80% methanol:water (LC-MS grade methanol, Fisher Scientific) at -70°C using a Tissue lyser cell disrupter (Qiagen). The extraction mixture was incubated at 4°C for 10 min, and then centrifuged for 5min at 13.2k rpm to separate the protein pellet.

The extraction procedure was repeated twice. The supernatants were pooled, dried in a speed-vac (Savant) and stored at −80°C. The pellets were solubilized in 200 µl of 0.2M NaOH by incubating at 95°C for 20 min, and protein contents were quantified with BioRad DC protein assays. Embryo metabolites were normalized to protein for LC/MS untargeted stable isotope tracing analysis.

Embryo extracts were analyzed by LC/MS as described previously (Chen et al., 2012; Ismailoglu et al., 2014) using a platform comprised of an Agilent Model 1290 infinity liquid chromatography system coupled to an Agilent iFunnel 6550 Quadrupole time-of-flight MS analyzer. Chromatography of metabolites was performed using aqueous normal phase gradient separation, on a Diamond Hydride column (Microsolv, NJ, USA). Mobile phases consisted of: (A) 50% isopropanol, containing 0.025% acetic acid and 6 µM EDTA and (B) 90% acetonitrile containing 5 mM ammonium acetate and 6µm EDTA. The following gradient was applied: 0–1.0 min, 99% B; 1.0–15.0 min, to 20% B; 15.0–29.0, 0% B; 29.1–37 min, 99% B. The LC/MS data were acquired in both positive and negative ionization modes.

Stable isotope tracing of [1,2-^{13}C] glycine: We applied an in-house untargeted stable isotope tracing (USIT) workflow (1,2) using Agilent untargeted metabolite profiling software [MassHunter Qualitative Analysis7.0, MassProfinder 8.0 and MassProfiler Professional (MPP 14.0)]. Labelled metabolites were identified on the basis of differential abundance in embryos cultured in [1,2-^{13}C] glycine compared with [1,2-^{12}C] glycine supplemented serum. Selected metabolites were identified on the basis of previously curated isotopologues. The

USIT workflow calculates and corrects for the natural abundance of ^{13}C isotope in samples using data from genotype-matched embryos labelled with [1,2-^{12}C]glycine.

Burren,K.A., Mills,K., Copp,A.J., and Greene,N.D.E. (2006). Quantitative analysis of s-adenosylmethionine and s-adenosylhomocysteine in neurulation-stage mouse embryos by liquid chromatography tandem mass spectrometry. J. Chromatogr. B Analyt. Technol. Biomed. Life Sci. *844*, 112-118.

Cabreiro,F., Au,C., Leung,K.Y., Vergara-Irigaray,N., Cocheme,H.M., Noori,T., Weinkove,D., Schuster,E., Greene,N.D., and Gems,D. (2013). Metformin retards aging in C. elegans by altering microbial folate and methionine metabolism. Cell *153*, 228-239.

Cockroft,D.L. (1990). Dissection and culture of postimplantation embryos. In Postimplantation Mammalian Embryos: A Practical Approach, A.J.Copp and D.L.Cockroft, eds. (Oxford: IRL Press), pp. 15-40.

Pryor,S.E., Massa,V., Savery,D., Greene,N.D.E., and Copp,A.J. (2012). Convergent extension analysis in mouse whole embryo culture. Methods Mol. Biol *839*, 133-146.
